# Establishment of a Wheat Cell-Free Synthesized Protein Array Containing 250 Human and Mouse E3 Ubiquitin Ligases to Identify Novel Interaction between E3 Ligases and Substrate Proteins

**DOI:** 10.1371/journal.pone.0156718

**Published:** 2016-06-01

**Authors:** Hirotaka Takahashi, Atsushi Uematsu, Satoshi Yamanaka, Mei Imamura, Tatsuro Nakajima, Kousuke Doi, Saki Yasuoka, Chikako Takahashi, Hiroyuki Takeda, Tatsuya Sawasaki

**Affiliations:** Proteo-Science Center (PROS), Ehime University, 3 Bunkyo-cho, Matsuyama, Ehime 790–8577, Japan; Augusta University, UNITED STATES

## Abstract

Ubiquitination is a key post-translational modification in the regulation of numerous biological processes in eukaryotes. The primary roles of ubiquitination are thought to be the triggering of protein degradation and the regulation of signal transduction. During protein ubiquitination, substrate specificity is mainly determined by E3 ubiquitin ligase (E3). Although more than 600 genes in the human genome encode E3, the E3s of many target proteins remain unidentified owing to E3 diversity and the instability of ubiquitinated proteins in cell. We demonstrate herein a novel biochemical analysis for the identification of E3s targeting specific proteins. Using wheat cell-free protein synthesis system, a protein array containing 227 human and 23 mouse recombinant E3s was synthesized. To establish the high-throughput binding assay using AlphaScreen technology, we selected MDM2 and p53 as the model combination of E3 and its target protein. The AlphaScreen assay specifically detected the binding of p53 and MDM2 in a crude translation mixture. Then, a comprehensive binding assay using the E3 protein array was performed. Eleven of the E3s showed high binding activity, including four previously reported E3s (e.g., MDM2, MDM4, and WWP1) targeting p53. This result demonstrated the reliability of the assay. Another interactors, RNF6 and DZIP3—which there have been no report to bind p53—were found to ubiquitinate p53 *in vitro*. Further analysis showed that RNF6 decreased the amount of p53 in H1299 cells in E3 activity-dependent manner. These results suggest the possibility that the RNF6 ubiquitinates and degrades p53 in cells. The novel *in vitro* screening system established herein is a powerful tool for finding novel E3s of a target protein.

## Introduction

Protein ubiquitination plays crucial roles in numerous cellular processes, including cell growth, regulation of diverse signal transduction, and the development of disease [1–3]. The small protein ubiquitin covalently attaches to the lysine residues of target proteins through its C-terminal glycine, thereby changing protein stability, function, or localization [4,5]. The attachment of a single ubiquitin molecule to a substrate is called mono-ubiquitination. Furthermore, the conjugation of one ubiquitin with another through their seven lysine residues or N-terminal methionine forms a polyubiquitin chain, each of which has a distinct function. For example, the polyubiquitin chain through lysine 48 induces target protein degradation by 26S proteasomes [4], and the polyubiquitin chain through lysine 63 functions as a scaffold for protein-protein interaction to mediate signal transduction [2] or form a protein complex involved in DNA repair [6].

Ubiquitination is mediated by three enzymatic reactions. Initially, ubiquitin is activated by the ubiquitin-activating enzyme E1 in an ATP-dependent manner via the formation of a high-energy thioester bond between the C-terminal glycine residue of ubiquitin and a core cysteine residue of E1. The activated ubiquitin is then transferred to a core cysteine residue of the ubiquitin-conjugating enzyme E2. Then, both E2 and the target protein bind to E3 ubiquitin ligase (E3), and E3 catalyzes the transfer of ubiquitin from E2 to the lysine residue of the target protein binding with E3 [4]. Therefore, the substrate specificity of protein ubiquitination is widely thought to be determined mainly by E3 [7]. To date, more than 600 E3s have been annotated in the human genome [8], and this diversity contributes to the specific recognition of numerous target proteins in eukaryotic cells. Identifying the E3 responsible for a target protein provides extensive information about the regulation mechanisms of half-life, localization, and functions in both the target proteins and the E3s in each biological phenomenon. In addition, interactions between E3s and their target proteins are considered attractive targets for drug discovery. For example, MDM2, an E3 that targets the tumor suppressor p53, is overexpressed in cancers such as sarcomas and leukemias [9,10]. Nutlin and its derivatives block the binding of MDM2 and p53 and protect p53 from proteasomal degradation, which results in cell death and the induction of apoptosis in many cancer-derived cell lines with intact p53 [11]. Moreover, chemical compounds targeting E3s such as Skp2 have been found to induce cell cycle arrest in various cancer cells [12]. Therefore, the identification of novel E3s targeting proteins of physiological significance has become a priority.

Assays based on living cells such as yeast, mammalian cultured cells, and model mouse are currently the primary tools used to identify combinations of E3s and their target proteins. Indeed, many physiologically important interactions between E3 and target protein were identified with various kinds of the cell-based assay [13–16]. However, these assays have some limitations. First, ubiquitinated proteins are usually degraded immediately by 26S proteasomes, and proteins ubiquitinated as non-degradative forms, such as K63, tend to shift to the insoluble fraction when cells are lysed. Second, some E3s are toxic to cells, further complicating the study of E3s with living cell assays. Third, the results from cell-based assays such as shRNA screening and immunoprecipitation from cell lysate often include indirect interactions. Therefore, the E3s of many target proteins—even widely studied proteins—remain unidentified.

Biochemical assays detecting direct binding between E3s and substrates by using recombinant proteins appear to be reasonable alternatives to assays using living cells because they exclude the possibility of low or absent expression of E3 or target proteins and the process of protein degradation in cells. These biochemical assays require the preparation of hundreds of active recombinant E3 proteins, but conventional protein-expressing systems such as *Escherichia coli* and insect cells have serious limitations. Furthermore, detecting the binding reactions of hundreds of proteins with general methods such as immunoprecipitation is extraordinarily difficult. To overcome these challenges, we used two methods in this study: a wheat germ-based cell-free protein synthesis system (wheat cell-free system), which efficiently produces many eukaryotic proteins including E3 in a 96-well format [17–19], and a high-throughput luminescence-based binding assay that is able to use crude protein samples (AlphaScreen). Combining these methods, we developed a novel *in vitro* screening assay using a protein array containing 250 E3s for the identification of novel interactions between E3 and their target proteins. Our preliminary screening with p53 found a high luminescence signal from interactions between p53 and known E3s such as MDM2 and MDM4. Novel E3s were also identified, and taken together, these results demonstrated that the assay developed herein is a powerful tool for the identification of novel E3s targeting proteins of interest.

## Results

### Preparation of the E3 protein array

**Fig 1:**
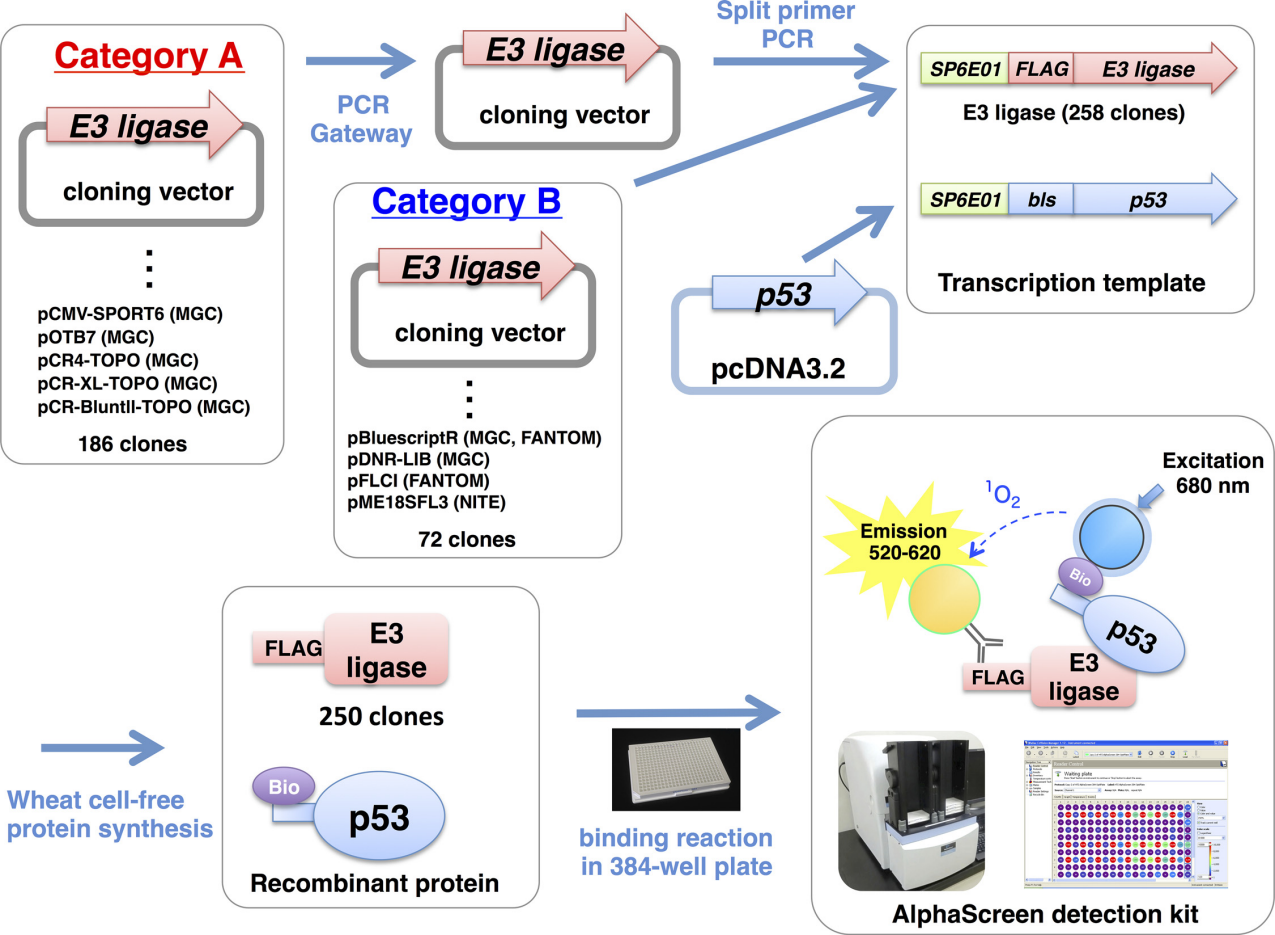
Study overview. cDNA clones encoding the E3 ubiquitin ligases (E3s) used in this study were divided into two categories. The clones in category B could be used directly for split-primer PCR, but those in category A were inserted into vectors that cannot be used directly for split-primer PCR and thus were transferred into the pDONR221 vector by using the Gateway system. The names of the vectors and the origins of the clones are indicated.

The E3s belonging to the single subunit E3 group such as HECT-type, U-box-type and many of RING-type are thought to recognize and ubiquitinate their target proteins without components other than E1 and E2 [8]. Another E3 group termed “multiple subunit E3” such as SCF- and APC-complex E3s requires multiple components for recognition and ubiquitination of target protein. In order to simplify the assay established here, we selected the single subunit E3 group as first trial and designed the assay to detect direct interaction between E3 and target protein so as to identify E3 for a target protein. At the beginning of this study, 232 human cDNAs encoding human E3 were selected from mammalian gene collection (MGC) clones covering more than 20,000 human full-length cDNAs without redundancy. In addition, cDNAs for three human and 23 mouse E3s not contained in the MGC clones were obtained from the NITE clone and FANTOM commercial full-length cDNA catalogs, respectively. Because many of the cDNAs in the MGC clones were inserted into backbone vectors unsuitable for the PCR to prepare transcription templates (the vectors in category A in Fig 1 and S1 Table), they were subcloned into pDONR221 by using Gateway technology. The detailed procedure and overview of the subcloning strategy are shown in Fig 1. Using these cDNAs, 258 DNA templates for *in vitro* transcription, including 241 RING-type, six U-box-type, and 11 HECT-type E3s, were prepared with a split-primer PCR method [17].

The recombinant E3 proteins were synthesized with the wheat cell-free system by using the robotic protein synthesizer GenDecoder [20]. The expression of each E3 was checked with immunoblot analysis, and 250 of the 258 E3s were confirmed to be synthesized by the system (S1 Fig).

### Model assay using p53 and MDM2

To establish the novel biochemical assay for identification of E3s for their target proteins, we selected the previously reported interaction between p53 and MDM2 as a model. This combination is one of the most widely studied target protein-E3 interactions. The transcription template of *MDM2* was obtained from the E3 cDNA library prepared in Fig 1. In addition, several reports have shown that cysteine 438 in the RING domain of MDM2 is critical for its E3 activity [21,22]. Therefore, a transcription template of *MDM2* in which cysteine 438 was replaced with serine (C/S) was prepared and used as a control MDM2 without E3 activity. The transcription template of *p53* was amplified by using the described split-primer PCR method. Furthermore, the transcription template of a mutant *p53* lacking the amino acids (residues 13–19) to interact with MDM2 (∆I) was also prepared and used as a negative control [23].

**Fig 2:**
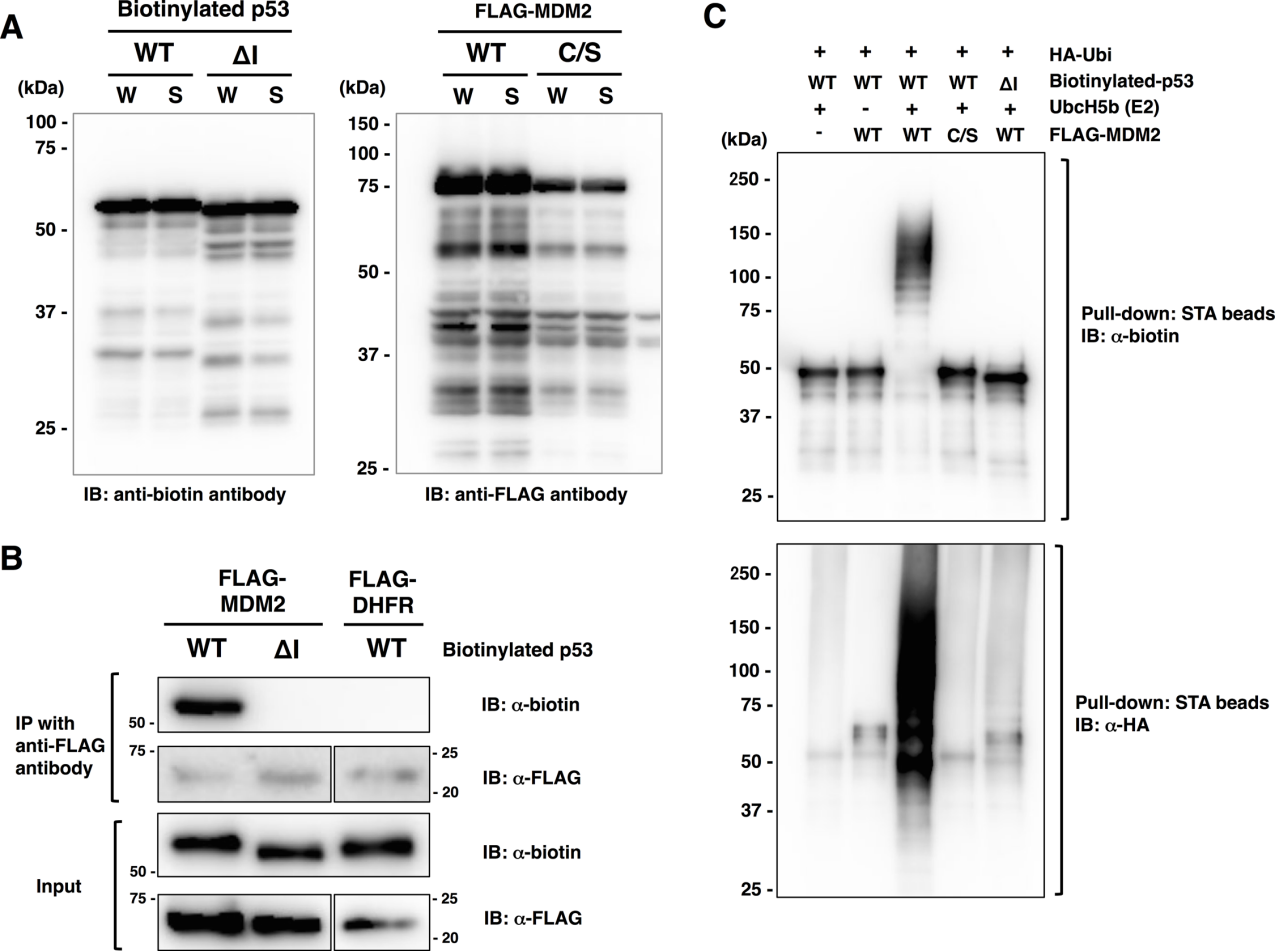
Model assay using p53 and MDM2. (A) Expression of recombinant proteins of p53 and MDM2 with the wheat cell-free system. Wild-type (WT) and mutant p53 lacking the amino acid residues required for binding with MDM2 (∆I) were synthesized as N-terminal biotinylated form. Wild-type MDM2 and its mutant with a single amino acid substitution in the catalytic core cysteine residue (C/S) were synthesized as N-terminal FLAG-tagged protein. Biotinylated and FLAG-dihydrofolate reductase (FLAG-DHFR) were used as negative controls for p53 and MDM2, respectively. The total translation mixture of each protein was centrifuged, and the whole translation mixture (W) and supernatant (S) were subjected to SDS-PAGE followed by immunoblot analysis (IB) using the antibodies indicated below. (B) Binding assay of biotinylated p53 and FLAG-MDM2 using a conventional immunoprecipitation (IP) assay. Fifteen microliters of crude biotinylated p53 (WT or ∆I) and FLAG-MDM2 were mixed and precipitated with anti-FLAG-antibody. Immunopreciptates were detected with anti-FLAG antibody and anti-biotin antibody. FLAG-DHFR was used as controls for FLAG-MDM2. (C) *In vitro* ubiquitination assay using biotinylated p53 and FLAG-MDM2. The crude translation mixtures of biotinylated p53 (WT or ∆I) and FLAG-MDM2 (WT or C/S) were mixed and then the reaction mixture containing ATP and HA-tagged ubiquitin (HA-Ubi) with or without UbcH5b was added. After the ubiquitination reaction, biotinylated p53 was pulled down with streptavidin (STA) magnet beads and subjected to SDS-PAGE followed by immunoblot analysis using anti-HA-antibody (for ubiquitin) and anti-biotin antibody (for p53).

The recombinant proteins of MDM2 and p53 were synthesized with the wheat cell-free system in an N-terminal FLAG-tagged form and an N-terminal single biotinylated form, respectively. As shown in Fig 2A, wild-type and ∆I mutant of p53 were synthesized successfully, and nearly identical amounts of recombinant p53 and MDM2 were observed in the whole translation mixture (W) and the supernatant (S) after the centrifugation of the former, which indicated that these proteins were synthesized in soluble forms. In addition, a conventional immunoprecipitation assay using anti-FLAG antibody showed that wild-type p53 but not ∆I was bound to MDM2 (Fig 2B).

To confirm that the binding between recombinant MDM2 and p53 shown in Fig 2A was a functional interaction, we performed an *in vitro* ubiquitination assay using the recombinant MDM2 and p53. As shown in Fig 2C, significant ubiquitination of p53 by MDM2 was observed only when biotinylated p53 was mixed with wild-type MDM2 but not C/S mutant of MDM2 or ∆I mutant of p53. These results indicated that the recombinant p53 and MDM2 synthesized with the wheat cell-free system functionally interacted in manner similar to that reported previously.

### Establishment of the high-throughput biochemical assay using AlphaScreen

**Fig 3:**
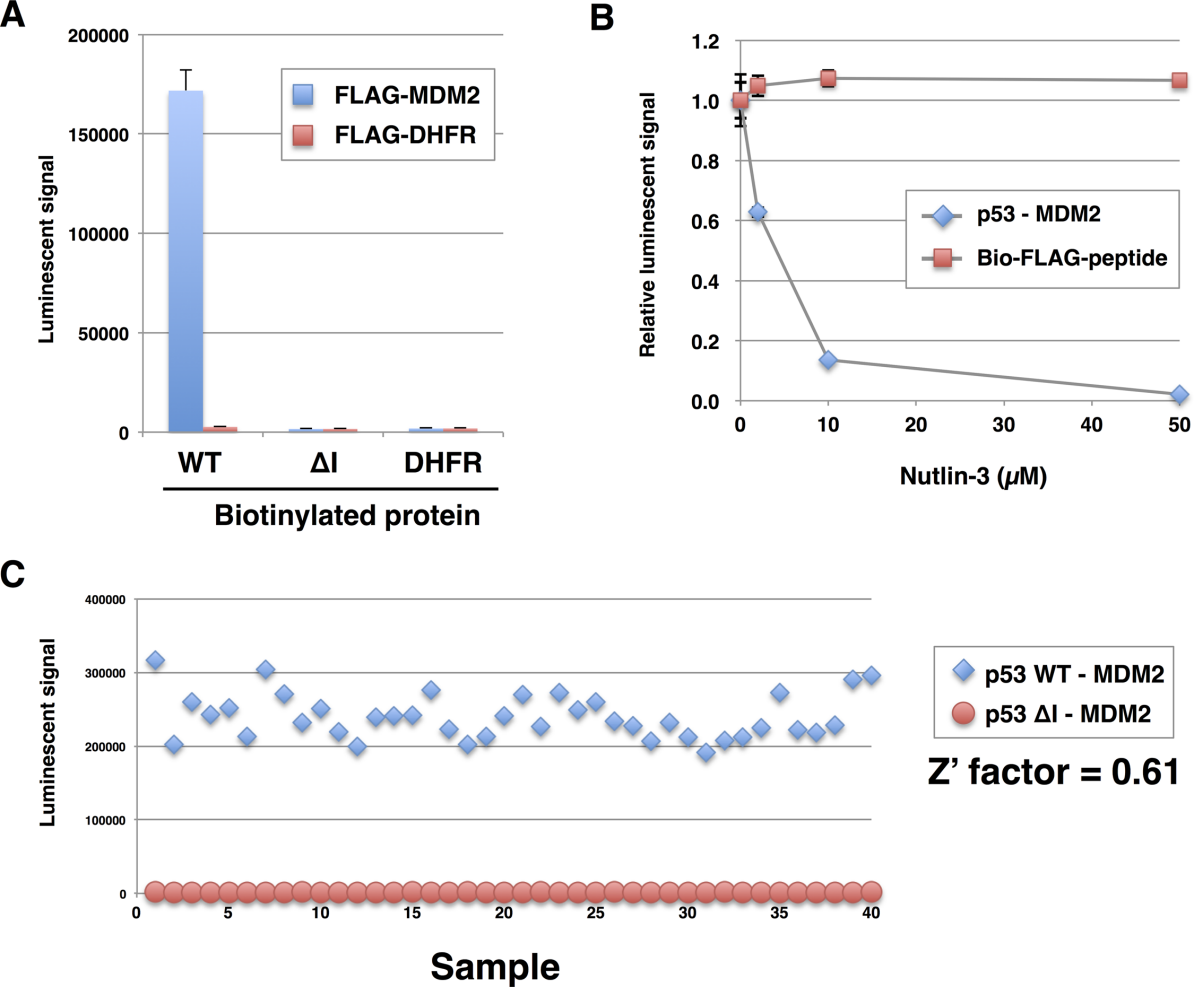
Development of AlphaScreen assay. (A) AlphaScreen assay to detect binding between p53 and MDM2. Crude translation mixtures of biotinylated p53 (WT or ∆I) and FLAG-MDM2 (0.75 μL each) were used for the assay. FLAG-DHFR was used as a control for MDM2. (B) A reaction mixture containing p53 and MDM2 prepared under the same conditions as those in (A) was mixed with 2, 10, or 50 μM Nutlin-3. Biotinylated FLAG-peptide was used as a control to measure the interference of Nutlin-3 in the AlphaScreen assay. (C) Validation of the quality of the AlphaScreen assay. Z′ factor was calculated from the reaction of wild-type p53 and MDM2 (positive control, n = 40, square) and the reaction of p53 ∆I mutant and MDM2 (negative control, n = 40, circle). In (A) and (B), all data are expressed as mean values of three independent experiments with error bars indicating standard deviations.

In order to identify E3s for a target protein from the E3 protein array, a first screening was designed to detect direct binding between E3s and target protein, because many of single subunit E3s we used in this study directly bind to target protein prior to ubiquitination. To establish the high-throughput binding assay, we used an AlphaScreen, a luminescence-based interaction assay, to detect interaction between recombinant MDM2 and p53. This assay is performed in a 384-well format with only small amount (0.5 to 1.0 μL) of the crude translation mixtures from the wheat cell-free system without purification [24,25]. As shown in Fig 3A, a high luminescence signal was observed when 0.75 μL of crude FLAG-MDM2 and wild-type biotinylated p53 were reacted. By contrast, only a background signal was obtained when either FLAG-MDM2 or biotinylated p53 were replaced by dihydrofolate reductase (DHFR), a negative control protein. In addition, ∆I mutant of p53 showed a faint signal even in the presence of FLAG-MDM2, which indicated that, similar to the immunoprecipitation assay (see Fig 2B), the AlphaScreen assay detected interaction between MDM2 and p53.

To further validate that the luminescence signal obtained in the AlphaScreen assay was an indicator of p53-MDM2 binding, we added Nutlin-3, a known small chemical compound inhibitor that blocks p53-MDM2 binding [11], to the AlphaScreen assay reaction. As expected, the luminescence signal decreased significantly with the addition of 2 μM Nutlin-3 and nearly disappeared when 50 μM was added (Fig 3B).

We also assessed the accuracy and quality of the AlphaScreen assay by calculating the Z′ factor. Assays with a Z′ factor greater than 0.5 are considered accurate and suitable for high-throughput screening [26]. Using 40 independent reactions containing biotinylated wild-type p53 and FLAG-MDM2 as the positive reaction and p53∆I and FLAG-MDM2 as the negative reaction, we obtained a Z′ factor of 0.61, which indicated that the assay was reliable.

### High-throughput binding assay to detect the binding between p53 and 250 E3s

We next screened p53-binding E3s from the protein array containing 250 E3s by using the constructed binding assay shown in Fig 3. Eight of E3s that the immunoblot analysis failed to detect (see S1 Fig and S1 Table) were also used for the binding assay as negative control. To subtract the background signal of each E3 obtained mainly from nonspecific binding between E3 and the detection beads or antibody, we also detected binding between all of the E3s and DHFR, the negative control. The value of the luminescence signal obtained from the reaction of E3 and biotinylated p53 was divided by the value of the reaction of E3 and biotinylated DHFR to yield a relative value indicating the binding activity of each E3 to p53.

**Fig 4:**
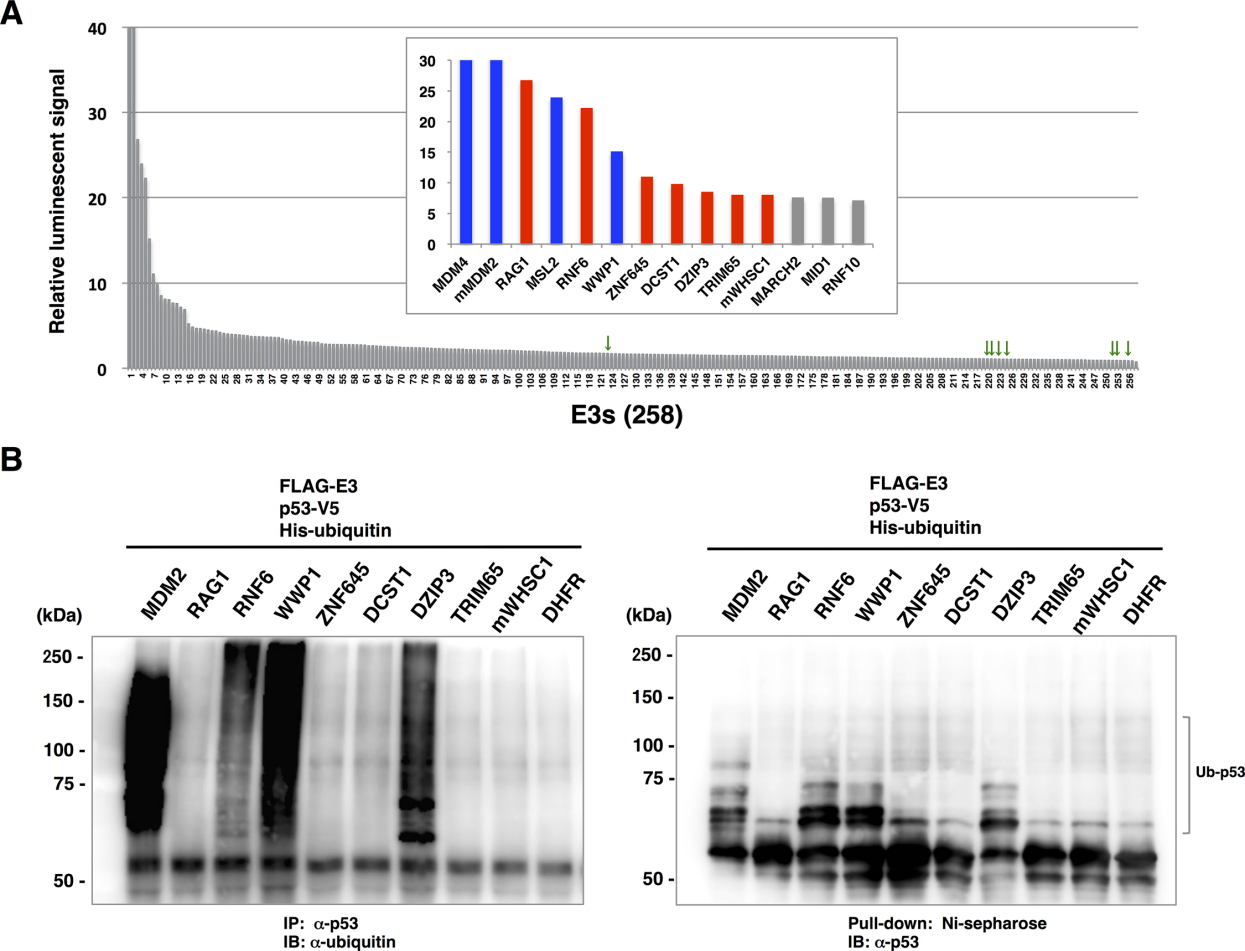
Comprehensive screening to identify p53-binding E3 ligases. (A) AlphaScreen assay to detect the binding between p53 and 258 E3s including eight negative controls. In this experiment, binding of all E3s to biotinylated p53 and biotinylated DHFR was measured, and the relative value was calculated as follows: value from E3 and p53 / value from E3 and DHFR. Green arrowheads indicated the negative controls. (B) *In vitro* ubiquitination assay using p53-binding E3s obtained in (A). p53-V5 was mixed with each E3, and the ubiquitination assay was carried out using His-ubiquitin. Left panel, p53-V5 was precipitated with anti-p53 antibody and detected with anti-ubiquitin antibody. Right panel, His-ubiquitin was pull-down with Ni-sepharose, and ubiquitinated p53 was detected with anti-p53 antibody.

The results of the binding assay demonstrated that in addition to MDM2, MDM4 gave an extremely high luminescence signal relative to those of the other E3s (Fig 4A). Except for these two E3s, nine E3s also showed a relative luminescence value of more than 8. Among these E3s, seven E3s such as RAG1 and RNF6 have not been reported to interact with p53, whereas MSL2 and WWP1 have been reported previously as E3s for p53 [27,28]. In contrast, all of the E3s that failed to observe their expression in S1 Fig showed only slight luminescent signal (Fig 4A, green arrowheads). The relative luminescent signals of all E3s were shown in S1 Table, and the E3s for which the relative luminescence signal was higher than 8 are listed in Table 1. Since the expression level of each crude recombinant E3 used in the binding assay was not equal (see S1 Fig), it was still unclear whether the various level of luminescent signal from individual E3s were due to the difference of the affinity of E3 to p53 or the amount of E3 protein. To solve this, MDM2, RAG1 and TRIM6 that showed highest, modest and low luminescent signal in Fig 4A, respectively, were selected and the binding assay was performed with various amount of these proteins. As shown in S2 Fig, MDM2 showed higher signal than RAG1 in all of four kinds of protein amount we tested. Even the luminescent signal obtained from the smallest amount of MDM2 (0.25 μl) was still much higher than that from the largest amount of RAG1 (3.0 μl). TRIM6 showed the lowest signal in all of protein amount The results of binding assay suggest that the luminescent signals reflect the affinity of each E3 to target protein rather the amount, at least to the amount applied shown in S2 Fig.

**Table 1:** Summary of hit E3 ubiquitin ligases (E3s). E3s for which the relative luminescence signals in Fig 4A were higher than 8 are listed. E3s indicated in bold character have been previously reported to interact with p53.

Rank	Gene name	Other name	GenBank accession No.	E3 type	Signal from DHFR	Signal from p53	relative value	References
1	**MDM4**	MDMX, HDMX, MRP1	BC105106	RING	998	196802	197.2	Shvarts *et al*. EMBO J (1996), 15, 5349–57
2	**mMDM2**	ACTFS, Hdm2, HDMX	AK088638	RING	1164	207070	177.9	Haupt et al. Nature (1997), 387:296–9
3	RAG1	RNF74	BC037344	RING	950	25466	26.8	
4	**MSL2**	MSL2-Like 1, KIAA1585, MSL2L1, RNF184, MSL-2	BC093790	RING	758	18152	23.9	Kruse and Gu, JBC (2009), 284:3250–63
5	RNF6	SPG2	BC034688	RING	1000	22270	22.3	
6	**WWP1**	Tiul1, AIP5, HSDRP1	BC036065	HECT	976	14778	15.1	Laine and Ronai, Oncogene (2007), 26:1477–83
7	ZNF645	HAKAIL, CT138	BC126190	RING	838	9254	11.0	
8	DCST1	-	BC064844	RING	854	8360	9.8	
9	DZIP3	HRUL138	BC063882	RING	824	7004	8.5	
10	TRIM65	-	BC013181	RING	952	7698	8.1	
11	mWHSC1	MMSET, NSD2, TRX5, REIIBP, WHS	AK038286	RING	842	6764	8.0	

To investigate whether the newly identified p53-binding E3s have E3 activity for p53, we subjected them to an *in vitro* ubiquitination assay. MDM2 and WWP1 were used as positive controls. The results showed that, in addition to the positive control E3s, RNF6 and DZIP3 ubiquitinated p53 (Fig 4B), although the intensity of p53 ubiquitination by MDM2 was much higher than that by other three E3s. These results suggested that RNF6 and DZIP3 are novel E3s targeting p53, and RNF6 that showed higher luminescent signal in Fig 4A was selected and used for further studies using cell-based assay.

### Cell-based assay to validate the results of the AlphaScreen assay

**Fig 5:**
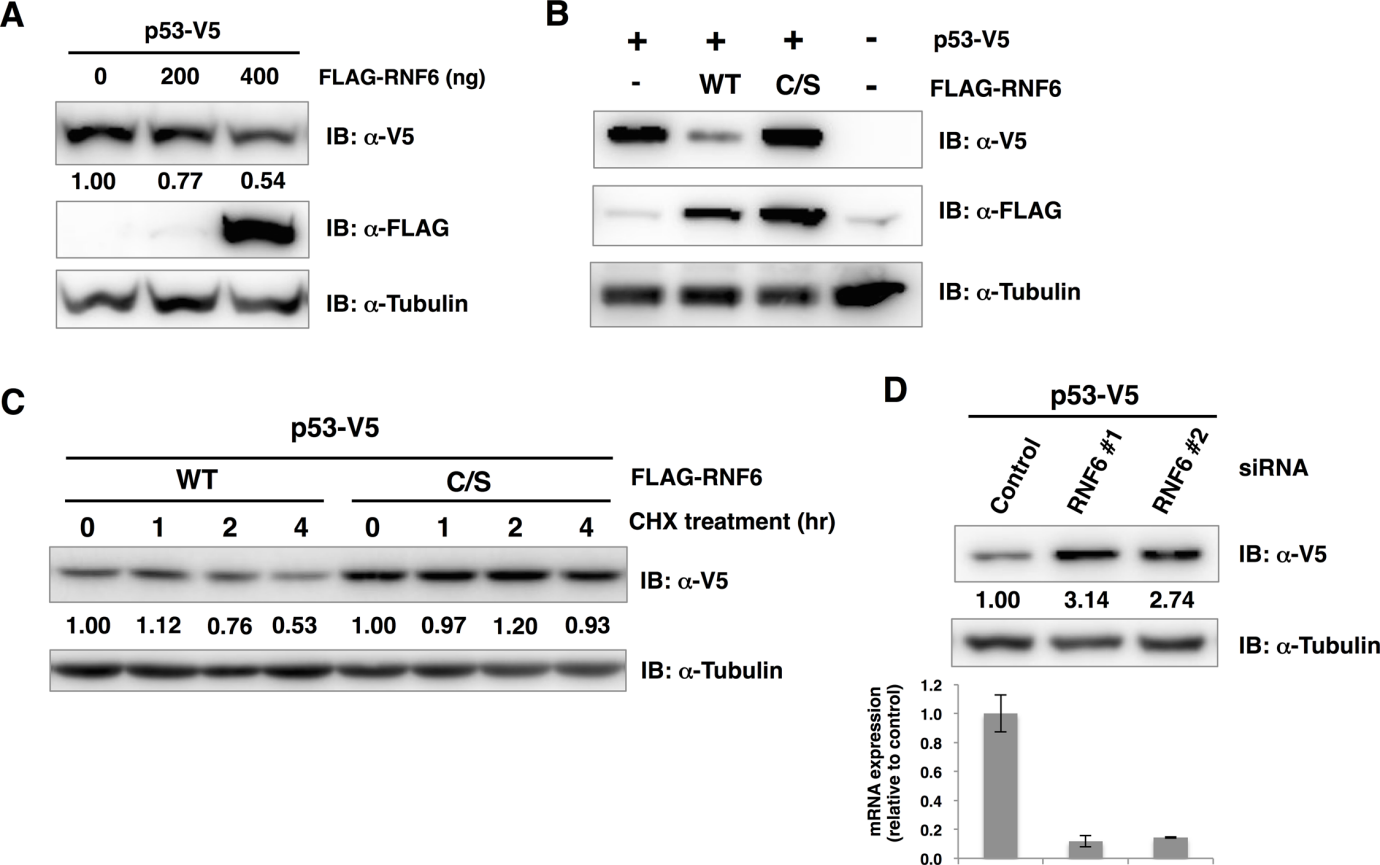
RNF6-dependent ubiquitination and degradation of p53 in cells. (A) Effect of RNF6 on p53 expression level. H1299 cells were co-transfected with a fixed amount of p53-V5 plasmid (25 ng) and an increased amount of FLAG-RNF6 plasmid (100 to 400 ng). Cell lysates from each reaction were subjected to SDS-PAGE followed by immunoblot analysis using anti-V5 antibody and anti-FLAG antibody. The band intensity of p53 in each reaction was quantified using imageJ software. Relative intensities normalized with the reaction of empty vector were indicated below the blot detected with anti-V5 antibody. (B) Co-transfection of p53-V5 with the wild-type (WT) or activity-deficient mutant (C/S) RNF6. (C) Stability of p53 in the presence of wild-type or C/S mutant of RNF6 was investigated by a pulse-chase assay. H1299 cells co-transfected with p53-V5 and wild-type or C/S mutant of FLAG-RNF6 were treated with 100 μg/ml of cycloheximide (CHX) for indicated time periods. The amount of p53-V5 detected by immunoblot analysis was quantified as same procedure as (A), and relative intensities normalized with the reaction without cycloheximide were indicated below the blot. (D) Endogenous RNF6 in H1299 cells were knockdown by siRNAs targeting RNF6, and overexpressed p53 was detected by immunoblot analysis (upper). The intensity of p53 band in each lane was quantified and intensites relative to the control were indicated below. Efficiency of RNF6 knockdown was confirmed with reverse transcription-quantitative PCR (lower).

The *in vitro* assays using recombinant E3s revealed that RNF6 bound to and ubiquitinated p53 (Fig 4A and 4B). Since one of the major roles of ubiquitination is thought to be the degradation of target protein by 26S proteasome activity, we speculated that RNF6 might ubiquitinate and degrade p53 in cell. To confirm this hypothesis, we co-transfected RNF6 and p53 into H1299 lung carcinoma cells to investigate the RNF6-dependent destabilization of p53. Initially, the expression level of p53 in the presence or absence of RNF6 was measured with an immunoblot analysis using the total cell lysate, which showed that the amount of p53 was decreased by RNF6 in a dose-dependent manner (Fig 5A). To investigate whether this decrease in p53 expression depends on E3 activity of RNF6, we substituted the RING domain cysteine residues at positions 632 and 635 with serine (C/S). This activity-deficient RNF6 mutant was co-transfected with p53 but did not affect the p53 expression level (Fig 5B). Then, we checked whether this decreased p53 expression level was due to reduction of stability of p53, a pulse-chase analysis was carried out using cycloheximide, an inhibitor of de novo protein synthesis. As a result, the amount of p53 was decreased in the presence of wild-type RNF6 after treatment with cycloheximide from two hours, whereas only slight change was observed in the presence of C/S mutant of RNF6 (Fig 5C), indicating that RNF6 down-regulated the expression level of p53 in a post-translational manner. Furthermore, effect of RNF6 knockdown on the amount of p53 was analyzed. Two small interfering RNAs (siRNAs) for RNF6 with different target sequences or control siRNA were transfected with H1299 cells. As a result, the amount of p53 was significantly increased by the knockdown of RNF6 but not by control siRNA (Fig 5D). These results, together with the result of *in vitro* ubiquitination assay shown in Fig 4B, suggested that RNF6 down-regulated the expression level of p53 in cells through ubiquitination of p53.

## Discussion

In this study, we developed a robust biochemical assay platform based on a protein array containing 250 recombinant E3 proteins and the high-throughput binding assay AlphaScreen. Using this assay, we identified some novel p53-binding E3s. Further analysis confirmed that RNF6 ubiquitinated p53 *in vitro* and down-regulated p53 expression in cells, which indicated the effectiveness of this assay platform for identifying novel E3 interactions with target proteins.

As for the high-throughput biochemical screening to identify the interaction between E3 and target protein, protein array/chip technology is often used. For example, some studies screened the target proteins of E3s using the protein chip containing thousands of yeast or human proteins, and identified many target proteins that were ubiquitinated by the E3s in cells. [29–31]. This report showed the effectiveness of the protein array technology. In this study, we aimed to identify novel E3 targeting protein of interest, therefore the protein array focused on E3s were prepared. Generally, the preparation of a large number of E3s is difficult with conventional expression systems such as those using *E*. *coli* and baculoviruses. However, our previous studies showed that the wheat cell-free system could synthesize active forms of recombinant proteins of RING-type and HECT-type E3s from the model plant *Arabidopsis* [24,32]. In the case of human E3, Tan *et al*. [33] prepared 72 purified E3s with the wheat cell-free system and identified a novel E3 involved in the integration activity of human immunodeficiency virus type 1. Although some E3s are self-ubiquitinated and degraded by 26S proteasomes in cells, the wheat cell-free system contains only negligible 26S proteasome activity, thereby allowing the stable expression of active E3s [24]. The results of these studies clearly show that the wheat cell-free system is suitable for the synthesis of E3 protein. Taking advantage of this system, therefore, we prepared a protein array containing hundreds of E3s for this study. Using commercial cDNA catalogs such as MGC, we prepared a total of 258 transcription templates containing 241 RING-type, six U-box-type, and 11 HECT-type E3s. After cell-free protein synthesis, immunoblot analysis confirmed that 250 of the 258 E3s (approximately 96%) were prepared (S1 Fig), indicating that this E3 protein array covered 75% of the single subunit E3 reported by Li *et al*. [8]. Furthermore, recombinant MDM2, WWP1, RNF6 and DZIP3 showed p53 ubiquitination activity on the *in vitro* ubiquitination assay (Fig 4B). Again, these results demonstrated the robustness of the wheat cell-free system for synthesis of E3, although the number of synthesized E3s that retained their activity remains unclear and it must be elucidated in a future study.

Using this E3 protein array, we aimed to identify E3 for a target protein of interest by detecting direct binding between E3 and target protein instead of ubiquitination of target protein by E3. Previously an *in vitro* assay to detect ubiquitination of target protein using AlphaScreen system was demonstrated [34], however, the purified E3 and target protein are required for this assay. Although we also have tried to detect ubiquitination of target protein by the AlphaScreen using crude recombinant proteins, the sensitivity was quite low probably because the E3 ubiquitinates not only substrate protein but the endogenous proteins in wheat germ extract. Moreover, the study by Tan *et al*. [33] demonstrated the *in vitro* screen using purified E3s prepared with wheat cell-free system, but 25% of the E3s could not be prepared properly. To consider the risk of purification, we based our assay platform on AlphaScreen technology that detects the binding interaction of E3s and substrate proteins by using crude translation mixture without purifications. In the AlphaScreen assay, donor beads and acceptor beads for detection specifically recognize biotinylated substrates and FLAG-tagged E3, respectively; therefore, specific interactions between E3 and its target protein in crude translation mixture are detected. We observed a high luminescence signal with slight background in our model study using the combination of MDM2 and p53, whereas these luminescence signals were not observed when mutant p53 lacking amino acids required for the binding with MDM2 was used (see Fig 3A). In addition, Nutlin-3, a small chemical compound inhibitor that blocks p53-MDM2 interaction, significantly decreased the luminescence signal observed with the p53-MDM2 combination (see Fig 3B). These results indicated that the binding assay established herein detected the p53-MDM2 interaction in a manner similar to those previously reported for *in vivo* and *in vitro* assays [11,23]. Our assay could also be carried out solely by mixing the crude proteins and reagents for detection (such as AlphaScreen beads and antibody) in a 384-well plate, and this simplicity in experimental procedure is another advantages of our assay.

The results of the comprehensive binding assay using 250 E3 proteins showed that compared with other E3s, two well-known E3s—MDM2 and MDM4—gave extremely high luminescence signals even though their expression levels were not much higher than those of the other proteins (S1 Fig). This result was consistent with the results of previous reports showing that MDM2 and MDM4 are the dominant binding partners of p53 in many cells [13,35,36]. In addition to MDM2 and MDM4, some previously reported E3s for p53 such as MLS2 and WWP1 resulted in high luminescence signals (Table 1), which indicated the reliability of our assay. Some of the other E3s known to target p53 did not show significant binding activity in the present study. These included the E3s identified with cell-based analyses such as immunoprecipitation, and it is possible that these E3s require additional cellular components for binding with p53. However, well-studied E3s such as RCHY1 (also called Pirh2) and COP1, for which direct binding to p53 has already been reported [37,38], showed only slight luminescence signals, even when the production of these E3s was confirmed (S1 Fig). This result might be attributable to the differences in the binding assays, the location of the tag on each protein, and requirements for post-translational modifications such as phosphorylation for the binding of E3 to the substrate.

The comprehensive screening in this study identified three novel p53 binding E3s, RAG1, RNF6 and DZIP3, and an *in vitro* ubiquitination assay showed that RNF6 and DZIP3 ubiquitinated p53. In addition, the expression level of p53 was decreased in H1299 cells when wild-type RNF6 but not C/S mutant of RNF6 was co-transfected. Although we carried out the cell-based ubiquitination assay to check the RNF6-dependent ubiquitination of p53 in cells, no significant ubiquitination of p53 was observed in the cells overexpressing wild-type RNF6. Considering the fact that RNF6 ubiquitinated p53 *in vitro* and decreased the stability of p53 in cells, however, it is possible that RNF6 ubiquitinates p53 in cells. The physiological functions of RNF6 are poorly understood, but recently one report showed that RNF6 was overexpressed in various leukemia cells, and induced proliferation of these cells. These results strongly indicated that RNF6 acts as an oncogene in these cells [39]. In this study, it was found that RNF6 bound to and degraded tumor suppressor p53 probably through ubiquitin/proteasome dependent manner. These results raise the possibility that RNF6-dependent p53 degradation is one of the triggers of canceration in these cells. In addition, another study reported that both RNF6 and MDM2 bind and ubiquitinate androgen receptors [40], suggesting that these two E3s function in close proximity and share some of the same target proteins in cells. Further analysis is required to elucidate the physiological significance of the RNF6-dependent ubiquitination and degradation of p53 in cells.

In conclusion, our protein array-based *in vitro* assay platform is a powerful tool for identifying novel E3s for target proteins. The assay enables not only the identification of the binding E3s of target proteins but also the performance of a ubiquitination assay that uses the same recombinant E3 protein. As a result of our screening using a p53 model, known E3s such MDM2 as well as an unknown E3 were identified as “hits,” which indicated that this assay is likely to be effective for identifying novel E3s targeting other proteins.

## Materials and Methods

### cDNA resources

We selected 232 human E3 cDNAs from the MGC clone (DNAFORM, Yokohama, Japan) and three from the NITE clone. In addition, 23 E3s for which commercial human cDNAs were unavailable were selected from the mouse cDNA catalog FANTOM (DNAFORM). The information of all E3 cDNAs used in the study is summarized in S1 Table. cDNAs of the mutant E3 with amino acid substitution and the ∆I mutant of p53 (deletion of amino acids 13 to 19) were prepared by using a PrimeStar mutagenesis basal kit (Takara bio, Otsu, Japan).

### Preparation of transcription templates for wheat cell-free protein synthesis

In order to prepare DNA templates of 258 *E3s* for *in vitro* transcription, these E3 cDNAs were fused with SP6 promoter, translation enhancer, and FLAG-tag sequences by split-primer PCR method [17]. The transcription templates of the bait proteins such as *p53* were fused with a biotin ligase recognition site in the N-terminus. In the case of the cDNA clones inserted in the vectors belonging to category A (see Fig 1 and S1 Table), the cDNAs were subcloned into pDONR221 vector by using the Gateway system (Invitrogen, Carlsbad, CA, USA), and the pDONR221-based constructs were used as templates for split-primer PCR.

### Wheat cell-free protein synthesis

The protein array containing the 258 E3s was produced by using the robotic synthesizer GenDecoder 1000 (CellFree Sciences, Yokohama, Japan) as described previously [20]. The biotinylated proteins were synthesized with a bilayer method that used 1 μL crude biotin ligase (BirA) produced by the wheat cell-free system and 500 nM D-biotin (Nacalai Tesque, Inc., Kyoto, Japan) [41]. To check the expression of each E3, we subjected 5 μL of the crude translation mixtures of all FLAG-fused and biotinylated proteins to SDS-PAGE followed by immunoblot analysis. For MDM2 and p53, the supernatant of the crude translation mixture after centrifugation at 22,000 × *g* for 10 min was also subjected to SDS-PAGE followed by immunoblot analysis to determine solubility. The FLAG-tagged and biotinylated proteins on the blot were detected by using horseradish peroxidase (HRP)-conjugated anti-FLAG antibody (M2, Sigma-Aldrich, St. Louis, MO, USA) and HRP-conjugated anti-biotin antibody (BN-34, Sigma-Aldrich), respectively.

### Immunoprecipitation

Twenty microliters of crude biotinylated protein and FLAG-tagged protein was mixed with a final concentration of 150 mM sodium chloride and incubated at 26°C for 1 h. The FLAG-tagged protein was then mixed with Protein G Dynabeads (Invitrogen) conjugated with anti-DYKDDDDK antibody (1E6, Wako Pure Chemical Industries, Ltd. Osaka, Japan) at 16°C for 1 h with rotation. After one hour incubation, the supernatant was removed by capturing the beads with a magnetic stand, and the beads were washed three times with phosphate-buffered saline buffer. Then, the FLAG-tagged protein was recovered from the beads with elution buffer containing 50 mM Tris-HCl, pH 7.5, 150 mM NaCl, and 200 μg/mL DYKDDDDK peptide (Wako) at 4°C for 30 min with gentle shaking. The protein was subjected to SDS-PAGE followed by immunoblot analysis using HRP-conjugated anti-FLAG antibody and anti-biotin antibody.

### Binding assay using AlphaScreen

The binding interactions between biotinylated p53 and FLAG-tagged E3s were detected with AlphaScreen technology provided by PerkinElmer. Fourteen microliters of biotinylated protein mixture containing 100 mM Tris-HCl, pH 7.5, 1 mg/mL bovine serum albumin, 0.1% Tween 20, 100 mM NaCl, and 0.75 μL crude biotinylated protein was dispensed into a 384-well OptiPlate (PerkinElmer, Waltham, MA, USA). Then, 0.75 μL crude FLAG-protein was added to the individual wells of the OptiPlate containing the biotinylated protein mixture and the plate was incubated at 26°C. After one hour incubation, a detection mixture containing 100 mM Tris-HCl, pH 7.5, 1 mg/mL bovine serum albumin, 0.1% Tween 20, 100 mM NaCl, 0.2 μg/mL Anti-DYKDDDDK antibody (1E6, Wako), 0.1 μL streptavidin-coated donor beads, and 0.1 μL protein A-conjugated acceptor beads was added to each well of the OptiPlate and the plate was incubated at 23°C for 1 h. Luminescence was analyzed with the AlphaScreen detection program. For the screening with the E3 protein array, biotinylated bait proteins were dispensed to OptiPlate by a FlexDrop dispenser (PerkinElmer) and then each FLAG-tagged E3 in 96-well titer plate was mixed with the biotinylated protein in the OptiPlate using Janus dispenser (PerkinElmer).

### *In vitro* ubiquitination assay

For checking the ubiquitination of p53 by wild-type or mutant of MDM2, twenty microliters of crude biotinylated p53 protein and 10 μL crude FLAG-MDM2 (wild type or C/S mutant) were mixed and incubated for 30 min at room temperature. Then, 10 μL of reaction mixture containing 80 mM Tris-HCl, pH 7.5, 12 mM ATP, and 1.2 μM UbcH5b (Enzo Life Sciences, Farmingdale, NY, USA), 5 μM HA-ubiquitin (boston biochem) was added to the crude protein mixture, and the reaction was incubated at 30°C for 3 h. After incubation, 10 μL of Dynabeads M-280 streptavidin (Invitrogen) resuspended with phosphate-buffered saline was added to each reaction and incubated for 1 h with rotation. The beads were then washed four times with 500 μL RIPA buffer containing 50 mM Tris-HCl, pH 8.0, 150 mM sodium chloride, 0.5 w/v% sodium deoxycholate, 0.1 w/v% sodium dodecyl sulfate, and 1.0 w/v% NP-40 substitute. After a final washing, the beads were boiled with SDS sample buffer and subjected to SDS-PAGE followed by immunoblot analysis using anti-HA HRP antibody (3F10, Roche, Indianapolis, IN, USA) and anti-biotin HRP antibody (BN-34, Sigma-Aldrich).

In the case of the ubiquitination assay using nine E3s identified by the AlphaScreen assay, fifty microliters of crude C-terminal V5-tagged p53 and 30 μL crude FLAG-E3 were mixed. The ubiquitination reaction was performed as same procedure described above except using His-tagged ubiquitin instead of HA-tagged ubiquitin. For detection of ubiquitinated p53, the reaction was then mixed with 500 μl of a denaturing buffer containing 100 mM Na_2_HPO_4_/NaH_2_PO_4_, pH 7.5, 6M guanidine hydrochloride, 10 mM imidazole. Then the reaction was mixed with 20 μl of Ni-sepharose beads (50% slurry, GE-healthcare) at 4°C for at least three hour. After two times washing with 1 mL of the denaturing buffer, the Ni-sepharose beads were further washed twice with a buffer containing 1 mL of 25 mM Na_2_HPO_4_/NaH_2_PO_4_, pH 7.5, 1.5 M guanidine hydrochloride, 17.5 mM imidazole. The sepharose was then washed once with a buffer containing 1 mL of 25 mM Tris-HCl, pH 6.8, 20 mM imidazole. The His-tagged proteins were eluted by boiling the sepharose with SDS sample buffer and were subjected to SDS-PAGE, followed by immunoblot analysis using anti-p53 antibody (DO-1, Santa Cruz Biotechnology, Santa Cruz, CA, USA). For the detection of ubiquitin attached to p53, the reaction was mixed with RIPA buffer, and p53 was precipitated with Dynabeads protein G (Invitrogen) conjugated with anti-p53 antibody. The ubiquitin on the blot was detected with anti-ubiquitin antibody (P4D1, Santa Cruz).

### Co-transfection of p53 and RNF6 in H1299 cells

Using Gateway technology, we cloned the open reading frames of p53 and the E3s into pcDNA3.2 DEST and pCMV-FLAG as a C-terminal V5 fusion and an N-terminal FLAG construct, respectively. H1299 cells were cultured in RPMI1640 Glutamax medium supplemented with 10% fetal bovine serum, 50 units/mL penicillin, and 50 μg/mL streptomycin. Approximately 8 × 10^4^ H1299 cells were cultured in a 24-well plate and, after 24 h, were transfected with 25 ng pcDNA-p53-V5 and 100 to 400 ng pCMV-FLAG-RNF6 (wild type or C/S) by using TransIT-LT1 (Takara). After incubation for 48 h at 37°C with 5% CO_2_, the cells were harvested. In the case of pulse-chase experiment using cycloheximide, the cells were treated with 100 μg/ml of cycloheximide for one to four hours prior to the harvest. The cell pellet was boiled in SDS sample buffer (125 mM Tris-HCl, pH 6.8, 4% SDS, 10% glycerol, 25 μg/mL bromophenol blue, 10% mercaptoethanol) and subjected to SDS-PAGE followed by immunoblot analysis using anti-FLAG antibody (M2, Sigma) and anti-p53 antibody (DO-1, Santa Cruz Biotechnology).

### Knockdown of RNF6 by siRNA

Two siRNAs targeting the independent region of *RNF6* and control siRNA were purchased from Sigma-Aldrich. H1299 cells (8 × 10^4^ cells) in 24-well plate were transfected with 5 pmol of each siRNA using Lipofectamine RNAiMAX reagent (Thermo Fisher Scientific). After incubation for 8 to 10 hours, the medium was changed to new medium and the cells were transfected 25 ng of pcDNA-p53-V5 for 36 hours. Then the cells were harvested and used for SDS-PAGE followed by immunoblot analysis. The mRNA expression level of RNF6 was analyzed with reverse transcription-quantitative PCR using SYBR Green real time PCR kit (Thermo Fisher Scientific).

## Supporting Information

S1 Fig Expression of the E3 ubiquitin ligase (E3) protein array. (A) The 258 E3 initially synthesized were subjected to SDS-PAGE followed by immunoblot analysis using anti-FLAG antibody. The E3s that failed to synthesize in this study were indicated by red characters. The E3s that failed to obtain in initial synthesis but obtained later by preparing new transcription templates were indicated as blue characters, and the result of immunoblot analysis was shown in (B). All of the assays, three microliters of the crude translation mixture of E3s was used.
(PPTX)

S2 Fig AlphaScreen assay using different amount of the E3 proteins. (A) Fix amount of biotinylated p53 (0.75 μl) was mixed with 0.25 to 3.0 μl of FLAG-E3s, and the binding was detected with AlphaScreen with same procedure as Fig 3A. The results from all three E3s were shown in left panel, and the same result without MDM2 were in right panel. (B) The crude E3 proteins with same amount as (A) were detected with immunoblot analysis using anti-FLAG-antibody. The band intensity of each E3 was quantified and was normalized with the intensity of 0.25 μl MDM2.
(PPTX)

S1 Table List of E3 ubiquitin ligases E3s in the protein array. (XLSX)
