# Supplementary material for: Establishment of a Wheat Cell-Free Synthesized Protein Array Containing 250 Human and Mouse E3 Ubiquitin Ligases to Identify Novel Interaction between E3 Ligases and Substrate Proteins
Source: PLoS One. 2016 Jun 1;11(6):e0156718. doi: 10.1371/journal.pone.0156718 (PMC4889105; doi:10.1371/journal.pone.0156718)
Supplement: S1 Fig — (A) The 258 E3 initially synthesized were subjected to SDS-PAGE followed by immunoblot analysis using anti-FLAG antibody. The E3s that failed to synthesize in this study were indicated by red characters. The E3s that failed to obtain in initial synthesis but obtained later by preparing new transcription templates were indicated as blue characters, and the result of immunoblot analysis was shown in (B). All of the assays, three microliters of the crude translation mixture of E3s was used. (PPTX) [file pone.0156718.s001.pptx]

## Slide 1
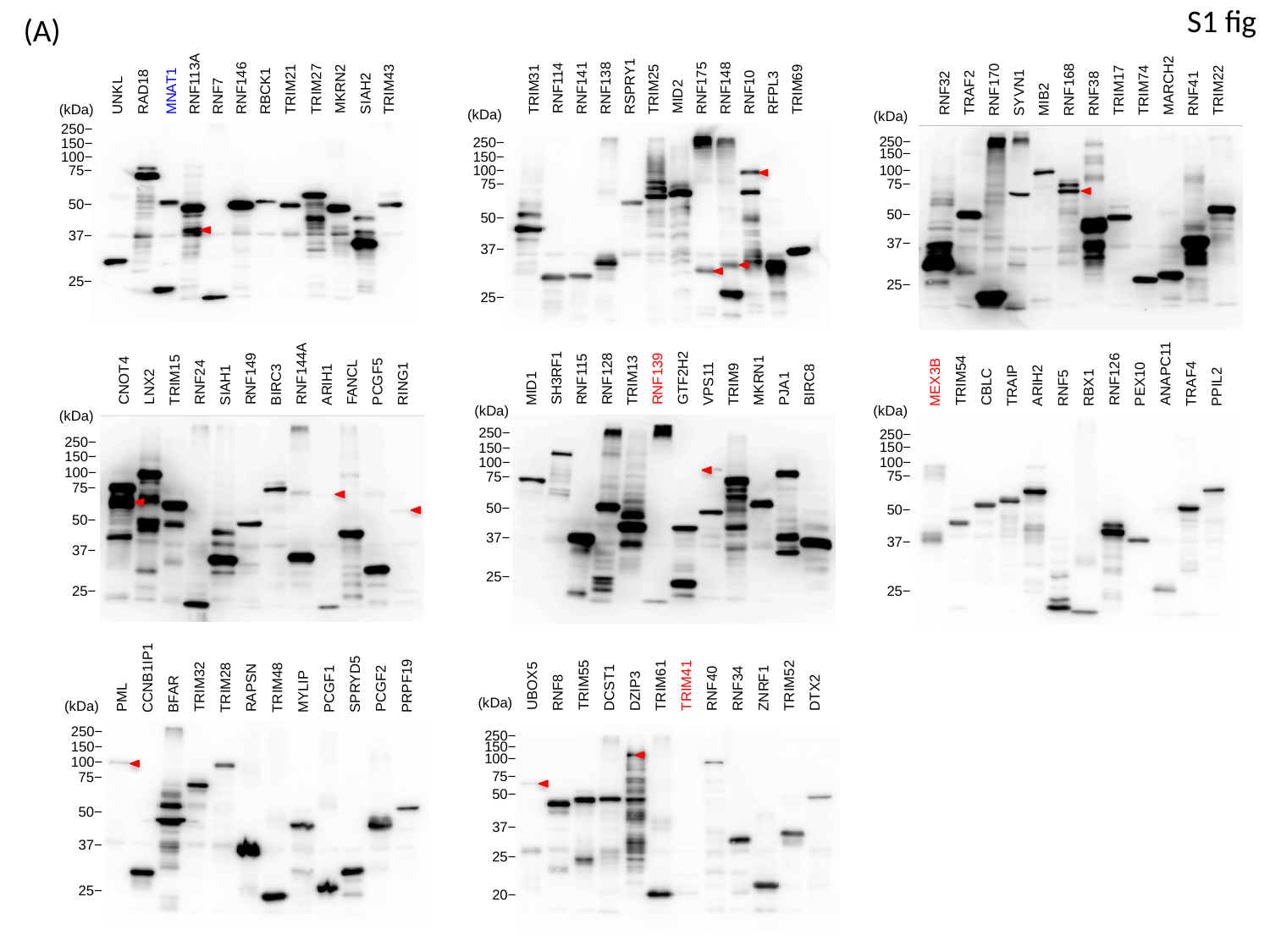

S1 fig
(A)
RNF113A
RNF146
TRIM27
MKRN2
TRIM21
TRIM43
RBCK1
MNAT1
RAD18
SIAH2
UNKL
RNF7
(kDa)
250−
150−
100−
75−
50−
37−
25−
MARCH2
RNF170
RNF168
TRIM22
TRIM17
TRIM74
SYVN1
RNF32
TRAF2
RNF38
RNF41
MIB2
250−
150−
100−
75−
50−
37−
25−
(kDa)
RSPRY1
RNF114
RNF141
RNF138
RNF175
RNF148
TRIM31
TRIM25
TRIM69
RNF10
RFPL3
MID2
250−
150−
100−
75−
50−
37−
25−
(kDa)
RNF144A
RNF149
TRIM15
CNOT4
PCGF5
RNF24
FANCL
RING1
ARIH1
BIRC3
SIAH1
LNX2
(kDa)
250−
150−
100−
75−
50−
37−
25−
ANAPC11
RNF126
TRIM54
MEX3B
TRAF4
PEX10
ARIH2
TRAIP
CBLC
PPIL2
RNF5
RBX1
(kDa)
250−
150−
100−
75−
50−
37−
25−
GTF2H2
SH3RF1
RNF128
RNF139
RNF115
TRIM13
MKRN1
VPS11
TRIM9
BIRC8
PJA1
MID1
(kDa)
250−
150−
100−
75−
50−
37−
25−
CCNB1IP1
SPRYD5
PRPF19
TRIM32
TRIM28
TRIM48
RAPSN
PCGF2
PCGF1
MYLIP
BFAR
PML
(kDa)
250−
150−
100−
75−
50−
37−
25−
TRIM55
TRIM41
TRIM61
TRIM52
UBOX5
DCST1
ZNRF1
RNF40
RNF34
DZIP3
RNF8
DTX2
(kDa)
250−
150−
100−
75−
50−
37−
25−
20−

## Slide 2
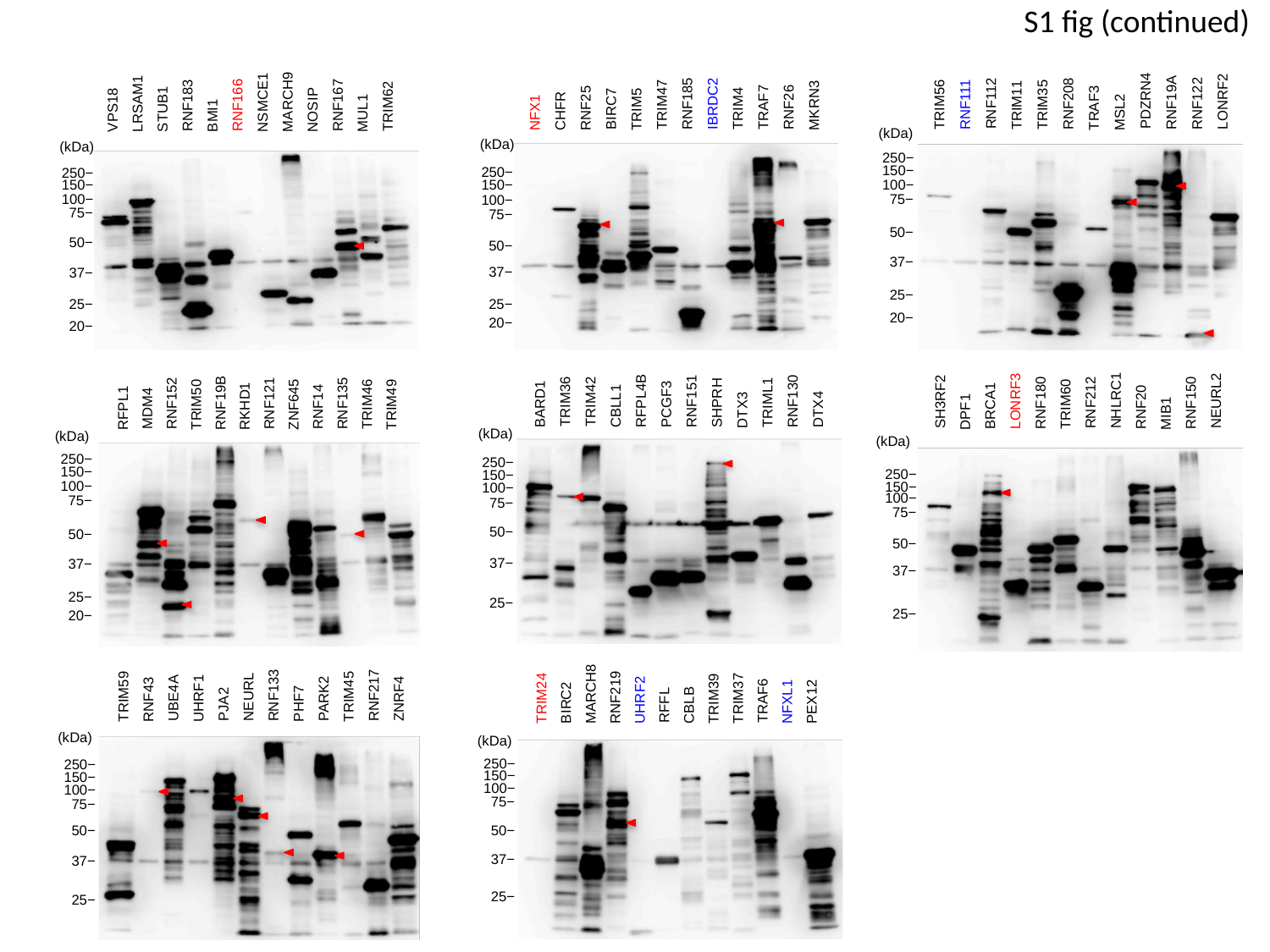

S1 fig (continued)
PDZRN4
LONRF2
RNF19A
RNF122
RNF112
RNF208
TRIM56
RNF111
TRIM35
TRIM11
TRAF3
MSL2
(kDa)
250−
150−
100−
75−
50−
37−
25−
20−
MARCH9
NSMCE1
LRSAM1
RNF166
RNF167
RNF183
TRIM62
STUB1
NOSIP
VPS18
MUL1
BMI1
(kDa)
250−
150−
100−
75−
50−
37−
25−
20−
IBRDC2
RNF185
TRIM47
MKRN3
TRAF7
RNF26
RNF25
TRIM4
BIRC7
TRIM5
CHFR
NFX1
(kDa)
250−
150−
100−
75−
50−
37−
25−
20−
NHLRC1
NEURL2
LONRF3
SH3RF2
RNF212
RNF180
RNF150
TRIM60
BRCA1
RNF20
DPF1
MIB1
(kDa)
250−
150−
100−
75−
50−
37−
25−
RFPL4B
RNF151
RNF130
TRIM36
TRIM42
TRIML1
SHPRH
BARD1
PCGF3
CBLL1
DTX4
DTX3
(kDa)
250−
150−
100−
75−
50−
37−
25−
RNF19B
RNF152
RNF121
RNF135
TRIM46
ZNF645
TRIM50
TRIM49
RKHD1
RNF14
RFPL1
MDM4
(kDa)
250−
150−
100−
75−
50−
37−
25−
20−
MARCH8
RNF219
TRIM24
TRIM37
TRIM39
UHRF2
TRAF6
PEX12
NFXL1
BIRC2
RFFL
CBLB
(kDa)
250−
150−
100−
75−
50−
37−
25−
RNF217
RNF133
TRIM59
TRIM45
NEURL
UBE4A
UHRF1
ZNRF4
PARK2
RNF43
PHF7
PJA2
(kDa)
250−
150−
100−
75−
50−
37−
25−

## Slide 3
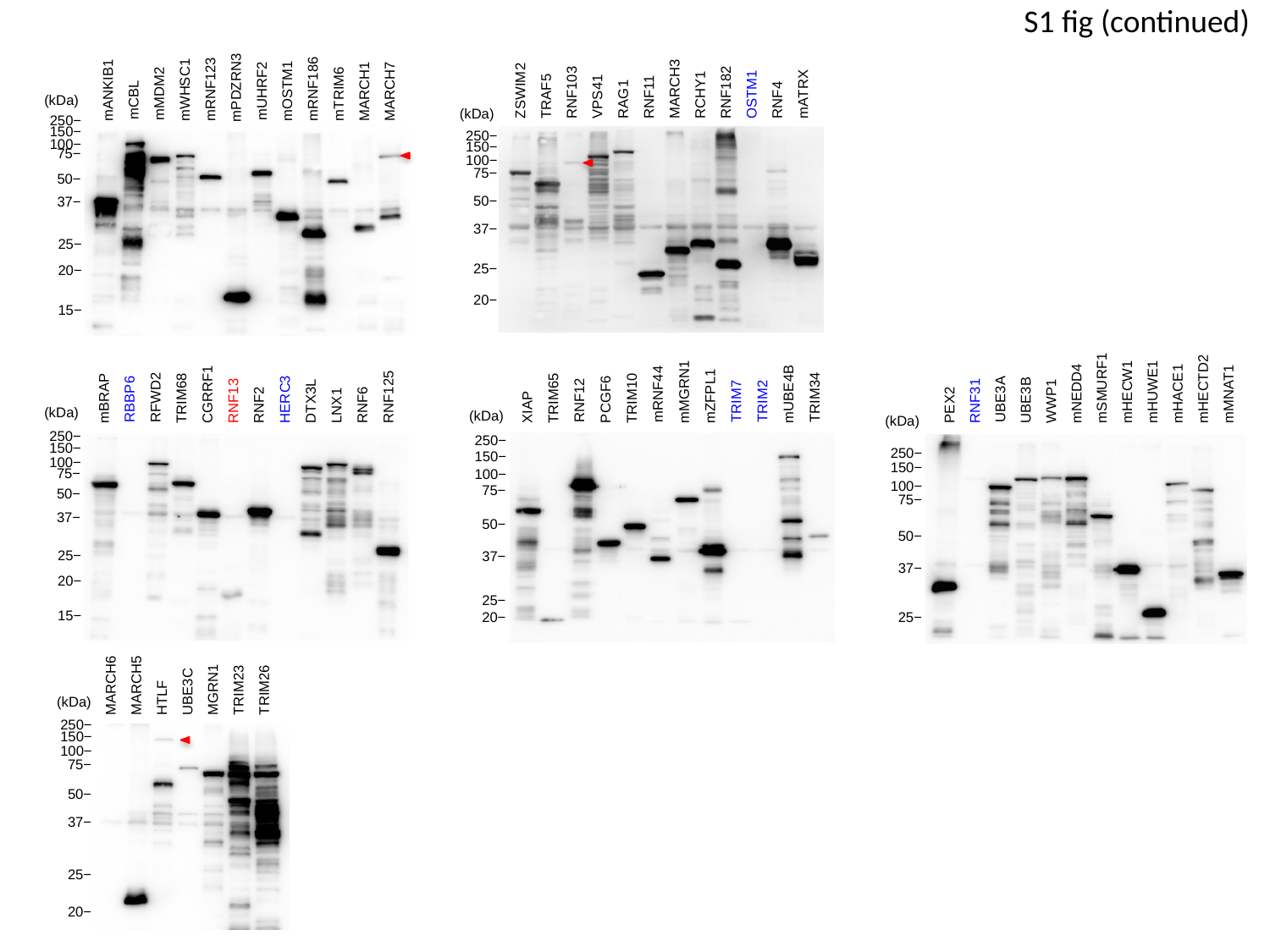

S1 fig (continued)
mPDZRN3
mRNF186
mRNF123
mWHSC1
mANKIB1
mOSTM1
mUHRF2
MARCH7
MARCH1
mTRIM6
mMDM2
mCBL
(kDa)
250−
150−
100−
75−
50−
37−
25−
20−
15−
MARCH3
ZSWIM2
RNF182
RNF103
mATRX
OSTM1
RCHY1
TRAF5
VPS41
RNF11
RAG1
RNF4
(kDa)
250−
150−
100−
75−
50−
37−
25−
20−
mSMURF1
mHECTD2
mHECW1
mHUWE1
mNEDD4
mHACE1
mMNAT1
UBE3A
UBE3B
RNF31
WWP1
PEX2
(kDa)
250−
150−
100−
75−
50−
37−
25−
mMGRN1
mRNF44
mUBE4B
mZFPL1
TRIM34
TRIM65
TRIM10
PCGF6
RNF12
TRIM7
TRIM2
XIAP
(kDa)
250−
150−
100−
75−
50−
37−
25−
20−
CGRRF1
RNF125
RFWD2
TRIM68
mBRAP
HERC3
RBBP6
RNF13
DTX3L
RNF2
RNF6
LNX1
(kDa)
250−
150−
100−
75−
50−
37−
25−
20−
15−
MARCH6
MARCH5
TRIM26
MGRN1
TRIM23
UBE3C
HTLF
(kDa)
250−
150−
100−
75−
50−
37−
25−
20−

## Slide 4
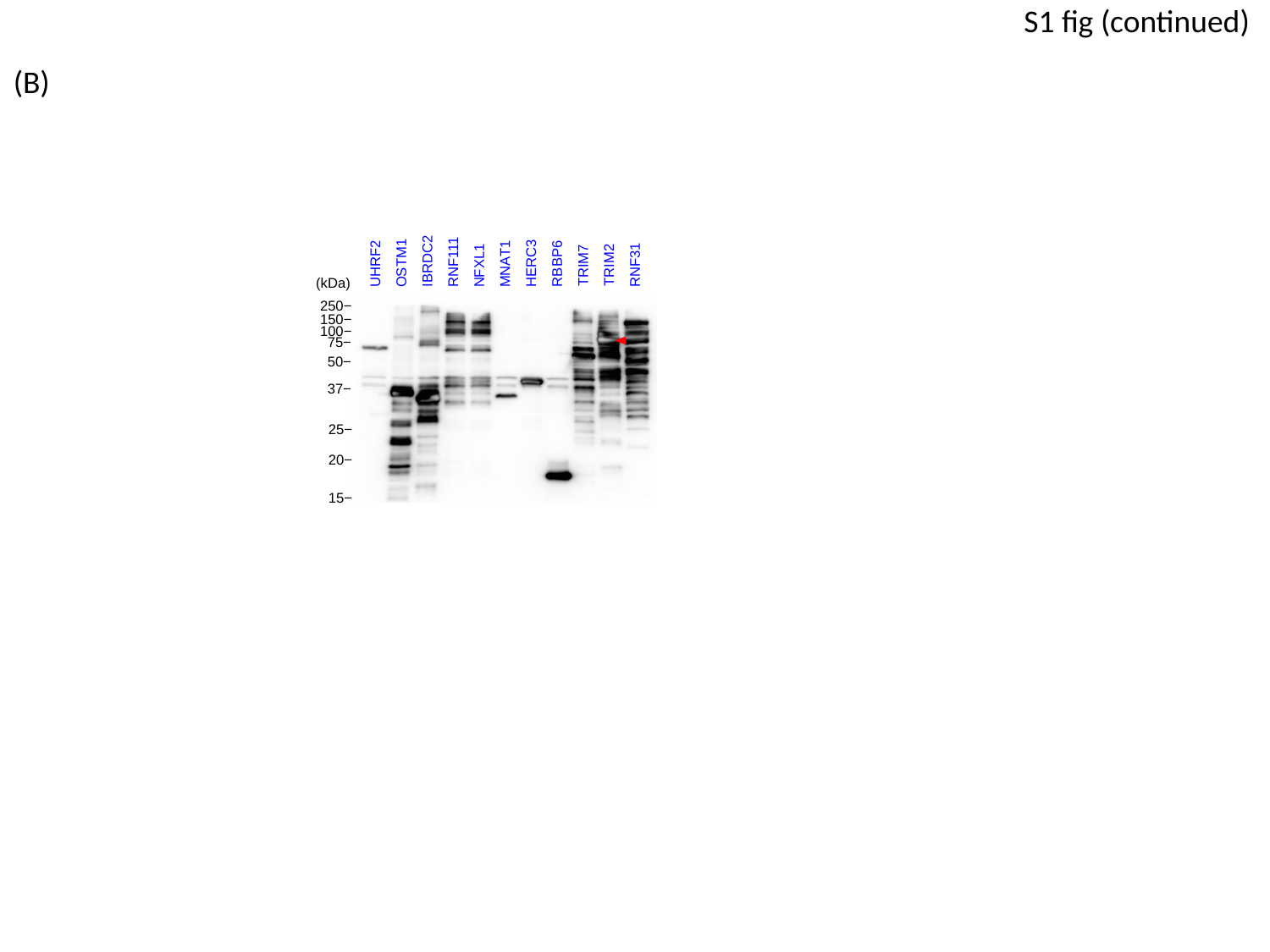

S1 fig (continued)
(B)
IBRDC2
RNF111
OSTM1
HERC3
UHRF2
RBBP6
MNAT1
RNF31
TRIM7
TRIM2
NFXL1
(kDa)
250−
150−
100−
75−
50−
37−
25−
20−
15−
