# Supplementary material for: Establishment of a Wheat Cell-Free Synthesized Protein Array Containing 250 Human and Mouse E3 Ubiquitin Ligases to Identify Novel Interaction between E3 Ligases and Substrate Proteins
Source: PLoS One. 2016 Jun 1;11(6):e0156718. doi: 10.1371/journal.pone.0156718 (PMC4889105; doi:10.1371/journal.pone.0156718)
Supplement: S2 Fig — (A) Fix amount of biotinylated p53 (0.75 μl) was mixed with 0.25 to 3.0 μl of FLAG-E3s, and the binding was detected with AlphaScreen with same procedure as Fig 3A. The results from all three E3s were shown in left panel, and the same result without MDM2 were in right panel. (B) The crude E3 proteins with same amount as (A) were detected with immunoblot analysis using anti-FLAG-antibody. The band intensity of each E3 was quantified and was normalized with the intensity of 0.25 μl MDM2. (PPTX) [file pone.0156718.s002.pptx]

## Slide 1
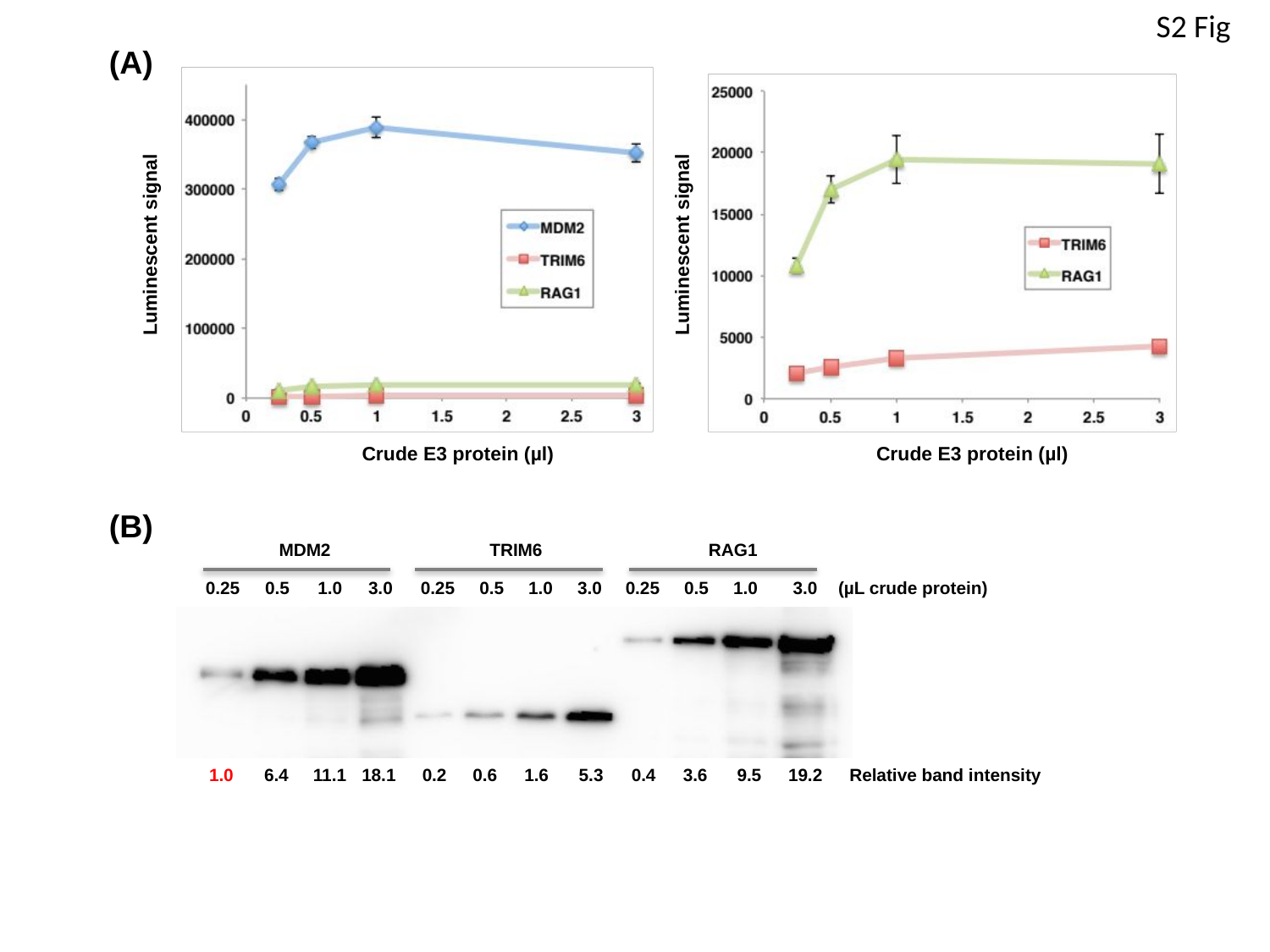

S2 Fig
(A)
Luminescent signal
Luminescent signal
Crude E3 protein (µl)
Crude E3 protein (µl)
(B)
MDM2
TRIM6
RAG1
(µL crude protein)
0.25
0.5
1.0
3.0
0.25
0.5
1.0
3.0
0.25
0.5
1.0
3.0
1.0
18.1
0.6
1.6
5.3
3.6
9.5
19.2
0.2
0.4
6.4
11.1
Relative band intensity
